# Chemopreventive Potential of Raw and Roasted Pistachios Regarding Colon Carcinogenesis

**DOI:** 10.3390/nu9121368

**Published:** 2017-12-18

**Authors:** Michael Glei, Diana Ludwig, Julia Lamberty, Sonja Fischer, Stefan Lorkowski, Wiebke Schlörmann

**Affiliations:** 1 Department of Nutritional Toxicology, Institute of Nutrition, Friedrich Schiller University Jena, Dornburger Straße 24, 07743 Jena, Germany; michael.glei@uni-jena.de (M.G.); diana-90@gmx.net (D.L.); julia_lamberty@hotmail.de (J.L.); sonja.d.m.fischer@gmx.de (S.F.); 2 Competence Cluster for Nutrition and Cardiovascular Health (nutriCARD), 07743 Jena, Germany; stefan.lorkowski@uni-jena.de; 3 Department of Nutritional Biochemistry and Physiology, Institute of Nutrition, Friedrich Schiller University Jena, Dornburger Straße 25, 07743 Jena, Germany

**Keywords:** apoptosis, colon cancer, dietary fiber, pistachio

## Abstract

Pistachios are rich in health-promoting bioactive compounds such as B vitamins, γ-tocopherol, polyphenols and dietary fiber, which could contribute to the reduction of colon cancer risk in terms of chemoprevention (Fischer, S.; Glei, M. Health-Potential of Nuts. Ernaehrungs Umsch. Int. 2013, 60, 206–215.). Since pistachios are often consumed roasted, the present study aims at investigating the influence of different roasting conditions (RC) on potential chemopreventive effects of pistachios in colon adenoma cells such as growth and apoptosis, genotoxic- and anti-genotoxic effects and modulation of gene expression of detoxifying enzymes (*CAT*, *SOD2*, *GPx1*, and *GSTP1*). Fermentation supernatants (FS) were obtained from raw and roasted (RC1 = 141 °C/25 min, RC2 = 160 °C/15 min and RC3 = 185 °C/21 min) pistachios after in vitro fermentation. FS of pistachios significantly reduced LT97 cell growth in a time- and dose-dependent manner. Compared to the blank control, pistachio FS (2.5%) led to a significant average reduction of H_2_O_2_-induced DNA damage (1.5-fold). Levels of *CAT* mRNA were significantly increased (1.3-fold, on average for 5% FS). Pistachio FS (5%) significantly increased the number of early apoptotic cells (up to 2.1-fold) and levels of caspase-3 activities (up to 6.9-fold). The present results confirm a chemopreventive potential of pistachios, which is mediated by growth inhibition, induction of apoptosis and anti-genotoxic effects, as well as induction of *CAT*. These effects remain mostly unaffected by roasting.

## 1. Introduction

Nuts, such as pistachios, are considered as health-promoting food, because of their high content of unsaturated fatty acids, dietary fiber, plant secondary metabolites, vitamins and micronutrients [1]. For example, a correlation between nut consumption and a reduced incidence of tumor diseases, such as cancers of colon, endometrium or pancreas [2], has been observed. The Nurses’ Health Study showed an inverse association between nut consumption and pancreatic cancer in women [3]. The same effect was found in the European prospective investigation into cancer and nutrition (EPIC) study for colon cancer [4], a cancer form whose incidence depends largely on the diet [5].

A possible reason for the observed effect against colon cancer could be the high content of dietary fiber in nuts. Fiber content ranges 5–12% depending on the type of nut [1], with the fiber content of pistachios being about 9% [6,7]. Different studies already demonstrated that dietary fiber can decrease risk for colon cancer [8,9]. This could be at least partially due to the fermentation of dietary fiber by the human gut microbiota, which results in the formation of short-chain fatty acids, e.g., butyrate [10]. Butyrate is already well recognized for its chemopreventive potential against colorectal cancer [11,12,13].

Furthermore, nuts are rich in bioactive substances such as vitamins, carotenes and phenolic substances [14]. In comparison to other nuts, pistachios contain high concentrations of thiamine, pyridoxine, γ-tocopherol and largely the carotenes lutein and zeaxanthin [14]. These might contribute to the chemopreventive potential against colon cancer, especially because of antioxidant properties [15,16].

Regarding the potential health effects of pistachios, it should be noticed that these are consumed mostly roasted [17]. The roasting process influences the taste, color and texture of pistachios and ensures microbiological safety [18,19,20]. Heat-treatment causes changes of the chemical structure of nut ingredients and formation of different metabolites such as Maillard reaction products. These generate the characteristic flavor and taste of roasted pistachios [21]. However, the roasting process may also affect health-promoting ingredients. Stuetz et al., for example, demonstrated an association between roasting and loss of β-carotene and tocopherols in pistachios [14]. In other studies, it was shown that the content of phenolic molecules in pistachios increased during roasting [20].

The aim of the present study was to investigate: (1) potential chemopreventive effects of pistachios regarding colon cancer by analyzing genotoxic and anti-genotoxic effects, cell growth inhibition and apoptosis as well as expression of genes involved in detoxification; and (2) the impact of roasting on these potential chemopreventive effects. Therefore, LT97 colon adenoma cells, which represent an early stage of colon carcinogenesis, were used to determine the impact of differentially roasted and in vitro-digested pistachios on above mentioned chemopreventive markers.

## 2. Materials and Methods 

### 2.1. Roasting 

Pistachios were provided by Paramount Farms (Lost Hills, CA, USA) and roasted in charges of 7.5 kg at laboratory scale using a FRC-T.1 drum roaster (Probat, Emmerich am Rhein, Germany) as described previously [22,23] with the following roasting conditions (RC): RC1 = 141 °C/25 min; RC2 = 160 °C/15 min; and RC3 = 185 °C/21 min. Subsequently, pistachios were stored at 4 °C in hermetically sealed bags until use.

### 2.2. In Vitro Fermentation 

In vitro digestion and fermentation of 2 g of ground pistachios was performed as described recently [23]. Briefly, after incubation with α-amylase for 5 min and pepsin for 2 h (37 °C), pistachio samples were treated with an intestinal fluid containing pancreatin and oxgall (26 mg and 50 mg, respectively, in 5 mL of 11 mM bicarbonate buffer; pH 6.5) and dialyzed under semi-anaerobic conditions (6 h, 37 °C). After in vitro fermentation of pre-digested samples (24 h, 37 °C) using a feces inoculum obtained from at least three healthy donors, fermentation supernatants (FS) were obtained by centrifugation. A blank fermentation sample without pistachios, representing the pure feces inoculum, was used as negative control, while Synergy1^®^ (oligofructose-enriched inulin, Beneo, Mannheim, Germany) served as positive control. FS obtained from the blank (fermentation negative control), Synergy1^®^ (fermentation positive control), raw and roasted pistachio (RC1, RC2, and RC3) samples were generated and used for cell culture experiments, which are described in the following sections.

### 2.3. Cell Culture 

LT97 colon adenoma cells (a kind gift from Professor B. Marian, Institute for Cancer Research, University of Vienna, Vienna, Austria) represent an early stage of colon carcinogenesis. The cells were prepared from colon micro-adenoma of a patient suffering from hereditary familial polyposis [24]. Cell culture conditions were described in detail previously [25]. Cells from passages 3 to 26 were used for cell culture experiments. Mycoplasma tests were performed regularly using the MycoAlert™ Mycoplasma Detection Kit (Lonza, Cologne, Germany) according to the manufacturer’s instructions. LT97 cells were checked for authenticity recently via STR (short tandem repeat) profiling (Leibnitz Institute DSMZ, German Collection of Microorganisms and Cell Cultures, Braunschweig, Germany).

### 2.4. DAPI Assay

LT97 colon adenoma cells were treated with FS obtained from raw and roasted pistachios in concentrations of 2.5%, 5%, 10% and 20% for 24 h, 48 h and 72 h. To determine time- and dose-dependent effects of pistachio FS on the growth of LT97 cells, the DAPI (4′,6-diamidino-2-phenylindol) assay was used as described previously [23]. Furthermore, sub-toxic concentrations of FS (EC25 and EC50: 2.5% and 5% FS, respectively) were determined via nonlinear regression/one phase exponential decay from three independent experiments (GraphPad Prism^®^ version 5, GraphPad Software, San Diego, CA, USA).

### 2.5. Comet Assay

Potential genotoxic and anti-genotoxic properties of pistachio FS were analyzed via Comet assay as described previously [26]. Briefly, LT97 cells were harvested and washed with PBS. Cell number and viability were measured using a ViCell cell counter (Beckman Coulter, Krefeld, Germany). LT97 cells (0.4 × 10^6^) were treated with FS (2.5% and 5%) of raw and roasted pistachios and controls (blank, Synergy1^®^) for 1 h at 37 °C. Anti-genotoxic effects of pistachio FS were analyzed in 0.4 × 10^6^ FS-pre-treated (45 min, 37 °C) and H_2_O_2_-FS-co-incubated cells (75 µM, 15 min, 37 °C). PBS or H_2_O_2_ (75 µM, 15 min at 37 °C) served as negative and positive control, respectively. After washing with PBS, 0.2 × 10^6^ cells were mixed with low-melting agarose (0.7%, Biozym, Hessisch Oldendorf, Germany) and spread onto microscopic slides coated with normal-melting agarose (0.5%, Biozym). After lysis (10 mM Tris-HCl, 100 mM Na_2_EDTA, 2.5 M NaCl, 10% DMSO, 1% Triton X-100, pH 10, 60 min, 4 °C), alkaline unwinding (1 mM Na_2_EDTA, 300 mM NaOH, pH 13, 20 min, 4 °C) and electrophoresis (20 V, 300 mA, 0.79 V/cm, 20 min, 4 °C) were carried out. Slides were washed with PBS (3 min × 5 min) and DNA was stained with SYBR^®^ Green (Sigma Aldrich, Munich, Germany) to determine the degree of DNA damage (ZEISS Axiostar plus, Carl Zeiss Jena, Jena, Germany and Comet Assay IV, Perceptive Instruments, Suffolk, UK) as tail intensity (%) as means of sixty cells.

### 2.6. Isolation of Total RNA

LT97 cells were treated with FS obtained from raw and differentially roasted pistachios and fermentation controls in concentrations of 2.5% and 5% in cell culture medium as well as 4 mM butyrate (additional positive control for dietary fiber fermentation) for 24 h. Then, RNA was isolated using the RNeasy Plus Mini Kit (Qiagen, Hilden, Germany) according to manufacturer’s instructions. Concentrations of RNA were measured with a NanoDropND-1000 photometer (NanoDrop Technologies, Wilmington, DE, USA). The RNA integrity number (RIN) was determined using the Agilent RNA 6000 Nano Kit (Agilent Technologies, Santa Clara, CA, USA) and an Agilent 2100 Bioanalyzer (Agilent Technologies, Waldbronn, Germany) according to the manufacturer’s instructions. Only RNA samples with a RIN >9 were used for further experiments.

### 2.7. cDNA Synthesis and RT-qPCR

The SCRIPT Reverse Transcriptase Kit (Jena Bioscience, Jena, Germany) was used to obtain complimentary DNA via reverse transcription of 1.5 µg total RNA in a 20 µL reaction mix (42 °C, 50 min). The reaction was stopped at 72 °C for 15 min and remaining RNA was removed by RNaseH treatment at 37 °C for 20 min. For qPCR experiments, cDNA samples were diluted (1:50) in RNase- free water. The mRNA levels of antioxidant and phase II enzymes (*CAT*, *SOD2*, *GPx1* and *GSTP1*) were analyzed by qPCR as described previously [23]. 

### 2.8. Flow Cytometry and Caspase Assay

After treatment (12 h and 24 h) of LT97 cells with FS of raw and roasted pistachios and controls (blank, Synergy1^®^) in concentrations of 2.5% and 5% as well as butyrate (4 mM), early apoptotic cells were quantified via flow cytometry (Cell Lab Quanta™ SC MPL 1.0, Beckman Coulter) using the annexin V-FITC/7-AAD (fluorescein isothiocyanate/7-aminoactinomycin D) kit (Beckman Coulter) according to manufacturer’s instructions. Caspase-3 activity as marker of advanced apoptosis was analyzed in LT97 cells treated with pistachio FS (2.5% and 5%) and FS of the controls for 24 and 48 h as well as butyrate (4 mM) as described previously [23]. Relative caspase activity was calculated as fold change relative to the medium control, which was set to 1.

### 2.9. Statistical Analyses

Means and standard deviations of at least three independent experiments were calculated. Statistical differences were analyzed by one- or two-way ANOVA including Bonferroni post-test or Student’s *t*-test for comparison of two groups using GraphPad Prism^®^ version 5 for Windows (GraphPad Software, San Diego, CA, USA).

## 3. Results

### 3.1. Pistachio FS Induce Growth Inhibition

**Figure 1:**
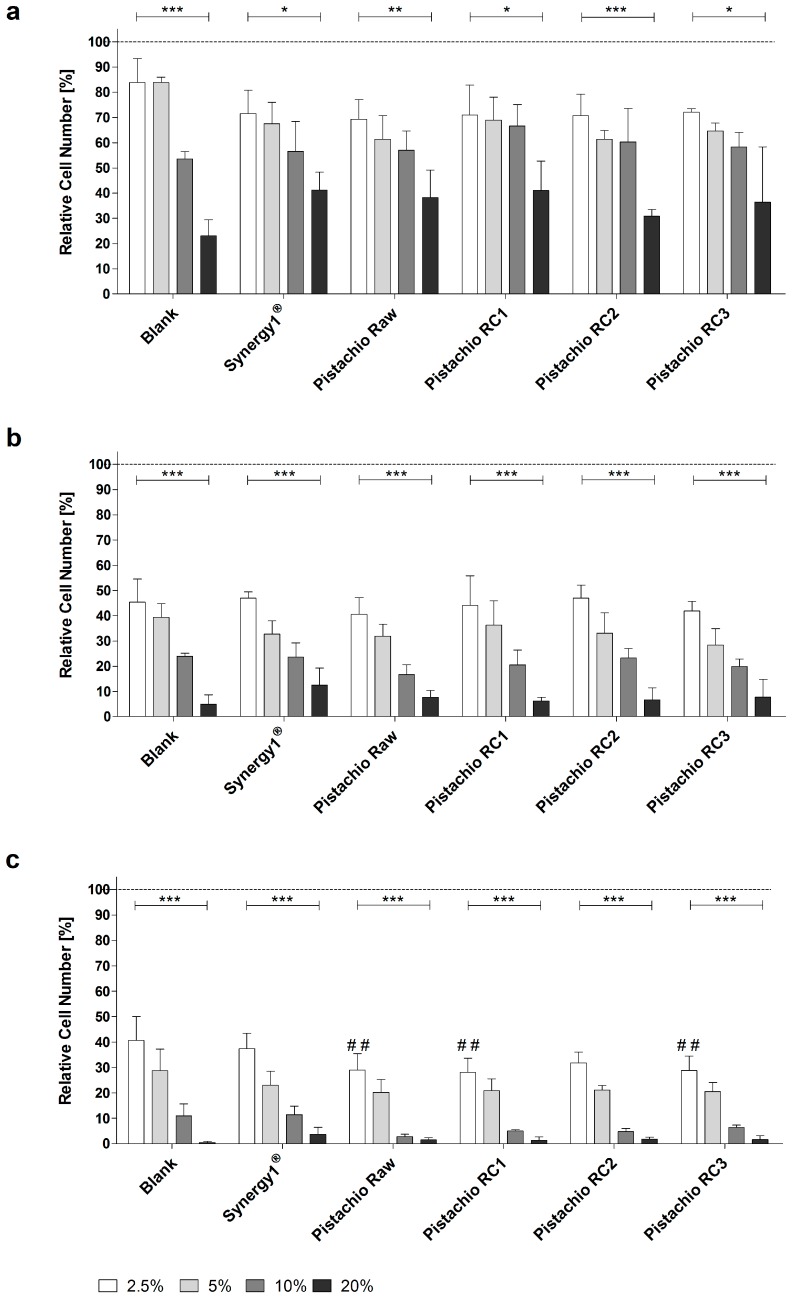
Growth inhibition of LT97 colon adenoma cells following incubation with fermented samples of raw and roasted pistachios (RC1 = 141 °C/25 min; RC2 = 160 °C/15 min; and RC3 = 185 °C/21 min) and controls (blank, Synergy1^®^) in concentrations of 2.5% to 20% for: (**a**) 24 h; (**b**) 48 h; and (**c**) 72 h (mean + SD, *n* = 4). Significant differences between blank and fermentation supernatants (FS) of Synergy1^®^ or pistachios (^##^ *p* ≤ 0.01) were obtained by two-way Anova/Bonferroni post-test. Significant differences between different concentrations (* *p* ≤ 0.05, ** *p* ≤ 0.01, *** *p* ≤ 0.001) were obtained by one-way Anova/Bonferroni post-test. All fermentation samples were significantly different compared to the medium control, which was set to 100% (dashed line). RC: roasting conditions.

Treatment of LT97 cells with FS obtained from pistachios as well as blank and Synergy1^®^ significantly reduced cell growth in a time- and dose-dependent manner in comparison to cells treated with medium. Average LT97 cell numbers ranged between 71.2 ± 8.9% and 33.2 ± 11.5% after treatment with 2.5% and 20% FS derived from pistachios and controls, respectively, for 24 h (Figure 1a).

After 48 h, cell numbers were reduced to 44.3 ± 6.5% and 7.7 ± 4.7% on average upon incubation with 2.5% and 20% FS, respectively (Figure 1b), while the same concentrations of FS resulted in LT97 cell numbers of 32.6 ± 7.3% and 1.7 ± 1.6% after 72 h (Figure 1c).

Important to note, FS obtained from raw and roasted pistachios (RC1 and RC3) reduced LT97 growth more effectively (down to 28.7 ± 0.5%, on average) than the blank control (40.7 ± 9.3%), particularly after 72 h and in a concentration of 2.5%. These growth inhibitory effects were independent from the roasting process. Concentrations of 2.5% and 5% FS (EC25 and EC50, respectively) were used for further experiments.

### 3.2. Pistachio FS Reduce H_2_O_2_-Induced DNA Damage 

**Figure 2:**
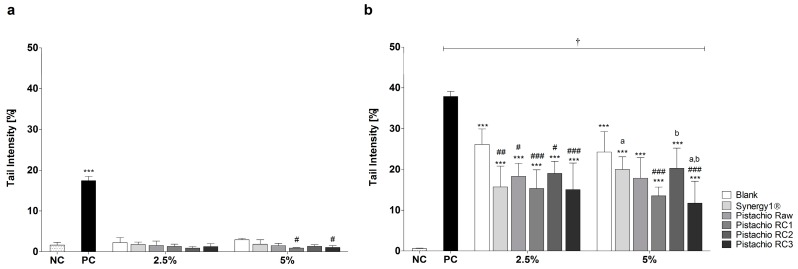
Genotoxic (**a**); and anti-genotoxic (**b**) effects of fermentation supernatants (FS, 2.5% and 5%) of raw and roasted pistachios (RC1 = 141 °C/25 min; RC2 = 160 °C/15 min; and RC3 = 185 °C/21 min) and controls (blank, Synergy1^®^) after treatment of LT97 colon adenoma cells for 1 h (mean + SD, *n* = 3). Significant differences between all FS and the positive control (PC, 75 µM H_2_O_2,_ *** *p* ≤ 0.001), the negative control (NC, PBS, ^†^ *p* ≤ 0.05) and the blank control (^#^ *p* ≤ 0.05, ^##^ *p* ≤ 0.01, ^###^ *p* ≤ 0.001) as well and between FS of raw and roasted pistachios and FS of Synergy1^®^ (^a,b^ *p* ≤ 0.05, equal letters represent significant differences) were obtained by two-way Anova/Bonferroni post-test. RC: roasting conditions.

Incubation of LT97 cells with FS obtained from pistachios and controls (blank, Synergy1^®^) resulted in average tail intensities of 1.5% (2.5% FS) and 1.6% (5% FS), which were comparable to the tail intensity of the negative control PBS (1.6%). A significantly higher tail intensity of 17.5% was measured after treatment with the positive control (75 µM H_2_O_2_). These results indicate that FS of pistachios and controls are not genotoxic. Furthermore, treatment with 5% FS of pistachios of RC1 and RC3 resulted in even significantly lower tail intensities compared to the blank control (Figure 2a).

After pre- and co-incubation of LT97 cells with FS obtained from pistachios and fermentation controls and 75 µM H_2_O_2_, tail intensities were significantly higher than the tail intensity of the negative control (Figure 2b). The negative control showed a tail intensity of 0.5%, while the tail intensity of the positive control (75 µM H_2_O_2_) was 37.9%. Treatment with 2.5% and 5% FS of the blank control resulted in a tail intensity of 26.1% and 24.3%, respectively. The tail intensities measured for Synergy1^®^ (FS 2.5%: 15.7% tail intensity, FS 5%: 20.0% tail intensity) were comparable to the tail intensities determined for pistachio FS (FS 2.5%: 16.9% tail intensity, FS 5%: 15.8% tail intensity, on average). Noteworthy, treatment of LT97 cells with pistachio FS as well as Synergy1^®^, particularly at concentrations of 2.5%, resulted in significantly lower tail intensities than the fermentation blank control, thus indicating that pistachio FS exhibit anti-genotoxic properties. No distinct differences of tail intensity measured after treatment with the FS obtained from pistachio of different RCs were observed.

### 3.3. Pistachio FS Modulate Gene Expression of CAT, SOD2 and GSTP1

**Figure 3:**
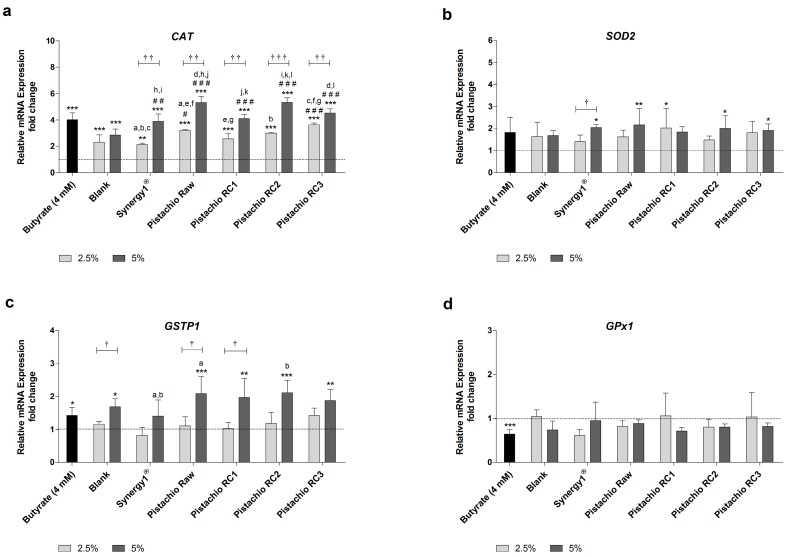
Relative mRNA expression of: (**a**) *CAT* (catalase); (**b**) *SOD2* (superoxide dismutase 2); (**c**) *GSTP1* (glutathione S-transferase P1); and (**d**) *GPX1* (glutathione peroxidase 1) in LT97 colon adenoma cells after incubation with fermentation supernatants (FS, 2.5% and 5%) of raw and roasted pistachios (RC1 = 141 °C/25 min, RC2 = 160 °C/15 min and RC3 = 185 °C/21 min) and controls (4 mM butyrate, blank, Synergy1^®^) for 24 h (mean + SD, *n* = 3). Values represent fold changes based on a medium control (set as 1, dashed line). Significant differences compared to the medium control (* *p* ≤ 0.05, ** *p* ≤ 0.01, *** *p* ≤ 0.001), to the blank control (^#^ *p* ≤ 0.05, ^##^ *p* ≤ 0.01, ^###^ *p* ≤ 0.001) and between FS obtained from raw and roasted pistachios and FS of Synergy1^®^ (^a–l^ *p* ≤ 0.05, equal letters represent significant differences) were obtained by two-way Anova/Bonferroni post-test or unpaired Student’s *t*-test (butyrate vs. medium control). Significant differences between 2.5% and 5% were obtained by unpaired Student’s *t*-test (^†^ *p* ≤ 0.05, ^††^ *p* ≤ 0.01, ^†††^ *p* ≤ 0.001). RC: roasting conditions.

Levels of *CAT* mRNA were significantly higher than the medium control upon treatment with all FS and butyrate as positive control (Figure 3a). Incubation with 2.5% and 5% FS obtained from raw and roasted pistachios resulted in a dose-dependent average increase of *CAT* mRNA levels of 3.1- and 4.8-fold, respectively. These upregulation of *CAT* mRNA expression was comparable to the induction caused by butyrate (4.0-fold) or FS derived from Synergy1^®^ (FS 2.5%: 2.1-fold, FS 5%: 3.9-fold), while the induction of *CAT* expression upon treatment with the blank control was lower (FS 2.5%: 2.3-fold, FS 5%: 2.8-fold). Importantly, treatment with pistachio FS, particularly at a concentration of 5%, resulted in significantly higher *CAT* mRNA levels compared to the blank control.

The induction of *SOD2* mRNA expression by pistachio FS was less pronounced than the induction of *CAT* gene expression (Figure 3b). FS obtained from Synergy1^®^ (FS 5%=2.0-fold), raw pistachios (FS 5%: 2.2-fold), RC1 (FS 2.5%: 2.0-fold), RC2 (FS 5%: 2.0-fold) and RC3 (FS 5%: 1.9-fold) led to significantly higher *SOD2* mRNA levels than medium (control), while the blank control or butyrate did not induce *SOD2* expression.

*GSTP1* mRNA levels were significantly induced in comparison to the medium control by 5% FS obtained from pistachios (2.0-fold, on average), the blank control (1.7-fold), and butyrate (1.4-fold) (Figure 3c). 

In contrast, *GPx1* mRNA expression was neither modulated by FS obtained from fermentation controls nor from raw and roasted pistachios (Figure 3d). Furthermore, even butyrate led to a significant down-regulation of *GPx1* mRNA levels (0.6-fold).

In general, the roasting of the pistachios did not affect the modulation of the gene expression of *CAT*, *SOD2*, *GSTP1* or *GPx1*.

### 3.4. Pistachio FS Induce Apoptosis 

**Figure 4:**
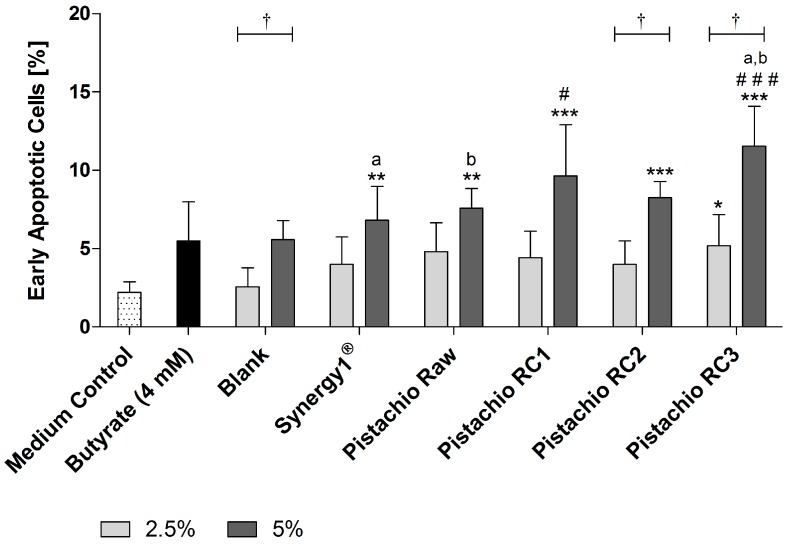
Number of early apoptotic LT97 cells in percent after incubation with fermentation supernatants (FS, 2.5% and 5%) of raw and roasted pistachios (RC1 = 141 °C/25 min; RC2 = 160 °C/15 min; and RC3 = 185 °C/21 min) and controls (4 mM butyrate, Synergy1^®^, blank) for 24 h (mean + SD, *n* = 3). Significant differences compared to the medium control (* *p* ≤ 0.05, ** *p* ≤ 0.01, *** *p* ≤ 0.001), to the blank control (^#^ *p* ≤ 0.05, ^###^ *p* ≤ 0.01) and between FS of raw and roasted pistachios and FS of Synergy1^®^ (^a,b^ *p* ≤ 0.05, equal letters represent significant differences) were obtained by two-way-Anova/Bonferroni post-test. Statistical significance of differences between 2.5% and 5% were calculated by unpaired Student’s *t*-test (^†^ *p* ≤ 0.05).

Detection of apoptosis by flow cytometry revealed that FS (5%) obtained from Synergy1^®^ (6.8 ± 2.2%) as positive fermentation control and pistachios (raw: 7.6 ± 1.3%, RC1: 9.6 ± 3.3%, RC2: 8.3 ± 1.0% and RC3: 11.5 ± 2.5%) were all able to significantly increase the number of early apoptotic cells in comparison to the medium control (2.2 ± 0.7%) after treatment for 24 h (Figure 4). In contrast, the respective blank control (5.6 ± 1.2%) and butyrate (5.5 ± 2.5%) did not increase the number of early apoptotic cells. Most importantly, treatment with FS obtained from roasted pistachios (RC1 and RC3) led to significantly higher levels of early apoptotic cells than the blank control. In addition, the induction of early apoptosis was significantly higher for FS of RC3-roasted pistachios than for raw ones.

**Figure 5:**
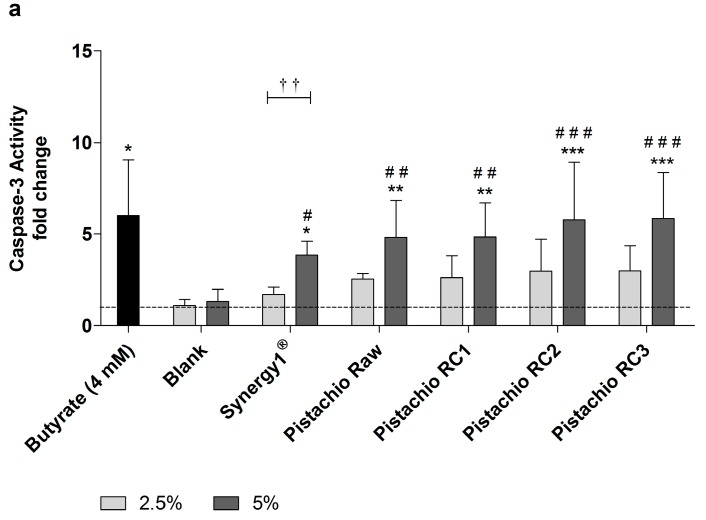

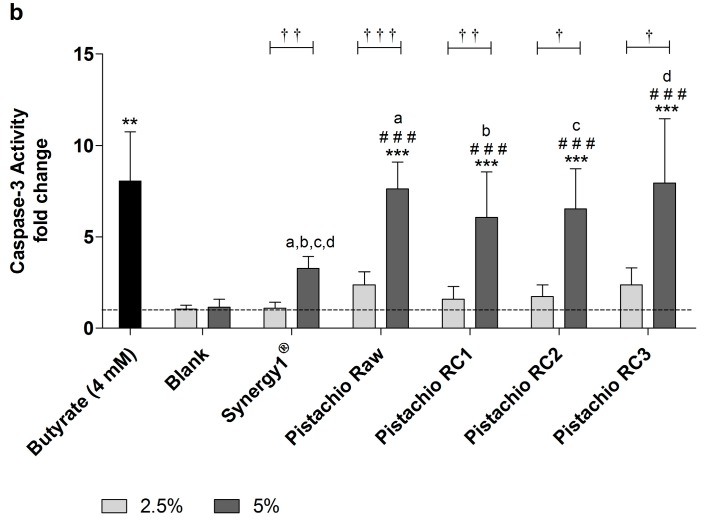
Caspase-3 activity in LT97 cells after incubation with fermentation supernatants (FS, 2.5% and 5%) of raw and roasted pistachios (RC1 = 141 °C/25 min, RC2 = 160 °C/15 min and RC3 = 185 °C/21 min) and controls (4 mM butyrate, Synergy1^®^, blank) for: (**a**) 24 h; and (**b**) 48 h (mean + SD, *n* = 4). Values represent fold changes based on a medium control (set as 1, dashed line). Significant differences compared to the medium control (* *p* ≤ 0.05, ** *p* ≤ 0.01, *** *p* ≤ 0.001), to the blank control (^#^ *p* ≤ 0.05, ^##^ *p* ≤ 0.01, ^###^ *p* ≤ 0.001) and between FS of raw and roasted pistachios and FS of Synergy1^®^ (^a–d^ *p* ≤ 0.05, equal letters represent significant differences) were obtained by two-way Anova/Bonferroni post-test or unpaired Student’s *t*-test (butyrate vs. medium control). Significant differences between 2.5% and 5% were obtained by unpaired Student’s *t*-test (^†^ *p* ≤ 0.05, ^††^ *p* ≤ 0.01, ^†††^ *p* ≤ 0.001). RC: roasting conditions.

In addition, caspase-3 activity as marker of advanced apoptosis was significantly enhanced upon treatment with 5% FS obtained from raw and roasted pistachios as well as Synergy1^®^ and butyrate compared to the medium and, more important, also to the blank control (Figure 5). In contrast, the lower concentration of 2.5% could not induce caspase-3 activity. After treatment of LT97 cells for 24 h with 5% FS or butyrate, the following relative caspase-3 activities could be measured (Figure 5a): butyrate (6.0 ± 3.0), blank (1.3 ± 0.7), Synergy1^®^ (3.8 ± 0.8), pistachios raw (4.8 ± 2.0) and roasted RC1 (4.8 ± 1.9), RC2 (5.8 ± 3.1), and RC3 (5.8 ± 2.5). After 48 h, caspase-3 levels were as follows (Figure 5b): Butyrate (8.1 ± 2.7), blank (1.1 ± 0.4), Synergy1^®^ (3.3 ± 0.7), pistachios raw (7.6 ± 1.5) and roasted RC1 (6.1 ± 2.5), RC2 (6.5 ± 2.2), and RC3 (7.9 ± 3.5).

Overall, no distinct impact of the roasting process on the induction of apoptosis in LT97 cells by FS was observed.

## 4. Discussion

Pistachios contain several health-promoting bioactive ingredients, such as polyphenols, B vitamins, γ-tocopherol, lutein and zeaxanthin as well as dietary fiber [6,14,27]. Due to this beneficial nutrient composition, the consumption of pistachios can contribute to the protection from different non-communicable chronic diseases. In general, results from intervention or prospective studies indicate that the consumption of nuts can reduce the risk for, e.g., cardiovascular diseases and total mortality [28,29], type II diabetes [30,31], or cancer [2,3]. There is evidence from the EPIC study that the consumption of nuts is associated with a reduced risk of colon cancer development [4]. How nuts can prevent colon cancer is not elucidated in detail. The relative high amount of dietary fiber could contribute to the colon cancer risk reducing effects since several studies have shown that the consumption of dietary fiber is inversely associated with colon cancer risk [9]. Pistachios contain up to 10 g dietary fiber per 100 g [6], which makes up for 10% of the daily recommended dietary fiber intake of 30 g [32], when considering the daily portion size of nuts (30 g) recommended by the WHO [33]. Pistachios are predominantly consumed roasted. The roasting process is responsible for the development of the typical taste of roasted nuts but leads to chemical and structural changes [18,34], which could affect bioactive compounds and potential chemopreventive effects. Therefore, the aim of the present study was to examine chemopreventive mechanisms of pistachios as a fiber rich food after an in vitro simulated fermentation and to elucidate the impact of different roasting conditions on these potential effects.

A hallmark of cancer chemoprevention is the inhibition of colon cancer cell growth [35]. The results from the present study show that pistachios can reduce number and growth of LT97 colon adenoma cells in a time- and dose-dependent manner. Similar results have been obtained in a previous study that investigated the chemopreventive potential of different raw nuts including pistachios [23]. In addition, the present results demonstrate that this growth inhibitory effect is not affected by the roasting process. It has been also shown that the fermentation of pistachios leads to the formation of butyrate [36], which is a potent inhibitor of colon cancer cell growth [37,38,39,40,41]. Therefore, this key fermentation product could be responsible for growth inhibition of LT97 cells. This could be due to the induction of apoptotic processes in colon cancer cells by butyrate [37,40,42,43]. Our study clearly revealed a pro-apoptotic action of pistachio FS on LT97 cells as the number of early apoptotic cells was increased and caspase-3 activity was induced. Therefore, induction of apoptosis might be at least one mechanism by which fermented pistachios or rather resulting metabolites such as butyrate exhibit growth inhibitory effects. The exact mechanism by which butyrate induces apoptosis in colon adenoma or cancer cells is not yet uncovered and many pathways are discussed, such as histone deacetylase inhibition [38,44,45], activation of the death receptor 5 [42], TGF-β_1_ [46], the JNK MAP [47] and mitochondrial pathways [43], as well as induction of the WNT pathway [48]. Importantly, FS represent complex mixtures of several nutritional compounds or fermentation metabolites of pistachios. Therefore, it cannot be excluded that other bioactive substances induce apoptosis, as it has been shown for urolithins which result from the microbial metabolization of phenolic compounds in the colon [49,50,51,52]. In addition, pistachios contain considerable amounts of selenium [22]. A portion of 30 g pistachios per day [33] can provide up to 6% of the recommended daily intake of selenium [32]. Selenium could contribute to the observed effects since it has been shown to exhibit growth inhibitory and apoptotic effects on colon cancer cells [53,54]. Furthermore, the number of early apoptotic cells was significantly increased exclusively by FS of roasted pistachios, especially by RC3. This might be due to a better accessibility and metabolization of apoptosis-inducing molecules after roasting.

Genotoxic effects of pistachio FS which could result in cell death could be excluded via Comet assay experiments. In contrast, fermented pistachios significantly reduced levels of H_2_O_2_-induced DNA damage in LT97 cells as indicated by lower tail intensities [55]. 

A reduction of potential carcinogens such as reactive oxygen species (ROS), which can cause DNA damage in colon cells is an initial step in colon cancer chemoprevention and can inhibit the initiation of cells as well as the progression of carcinogenesis [45]. This reduction could be due to antioxidant compounds in pistachios and pistachio FS, respectively, such as bioactive phytochemicals (e.g., phenolic acid, proanthocyanidins, flavonoids and γ-tocopherol), [6,14,27]. Although other studies indicated that phenolic compounds could be reduced upon roasting, as reviewed by Bolling et al. [56], no distinct influence of the roasting conditions regarding anti-genotoxic effects of pistachios could be observed in the present study. Noteworthy, results from a previous study of our group showed no loss of antioxidant activity of differentially roasted pistachios [22].

The induction of antioxidant active enzymes could also contribute to the reduction of DNA damage induced by ROS [45]. In the present study, particularly *CAT* expression was significantly enhanced by pistachio FS, whereas expression of *SOD2*, *GSTP1* and *GPx1* was not or only marginally affected. These results are in line with a recently published study investigating the chemopreventive potential of different nuts in LT97 colon adenoma cells [23]. In addition, the present study revealed no clear impact of the different roasting conditions of pistachios on *CAT* expression. Several mechanisms might be involved in the induction of *CAT* expression. Butyrate which was used as positive control also induced *CAT* mRNA levels, similar to pistachio FS. Previous studies also demonstrated that butyrate as a key fermentation product of dietary fiber [23,57] or FS obtained from other dietary fiber rich sources [40,58] induces *CAT* expression in colon adenoma or cancer cells. In addition, other dietary compounds of pistachios, such as flavonoids and polyphenols, might be involved in the induction of *CAT* expression by activating NF-E2-related factor 2 (Nrf2) via ARE (antioxidant response element) [59]. 

A limitation of our study is that significant effects were also caused by the blank control in some experiments. In addition to the other samples used in the present study, the blank control also contained dietary fiber as result of the nutritional habits of the feces donors. Although the used in vitro digestion and fermentation system is a good and widely accepted model to examine chemopreventive effects, it remains an artificial approximation of the situation in vivo. Another limitation might be the use of already transformed colon adenoma cells as in vitro model.

## 5. Conclusions

This is the first study investigating the impact of different roasting conditions on chemopreventive effects of pistachios. The results of the present study indicate that pistachios exhibit chemopreventive properties by inhibiting growth of adenoma cells, reducing levels of DNA damage and inducing *CAT* expression as well as apoptosis. In addition, the roasting process had no consistent or diminishing impact on these chemopreventive effects, which is an important finding, since pistachios are often consumed roasted.
